# Estimating the costs and cost‐effectiveness of HIV self‐testing among men who have sex with men, United States

**DOI:** 10.1002/jia2.25445

**Published:** 2020-01-20

**Authors:** Ram K Shrestha, Pollyanna R Chavez, Meredith Noble, Stephanie L Sansom, Patrick S Sullivan, Jonathan H Mermin, Robin J MacGowan

**Affiliations:** ^1^ Centers for Disease Control and Prevention National Center for HIV/AIDS, Viral Hepatitis, STD, and TB Prevention Atlanta GA USA; ^2^ Evidence Solutions Group Hayes Inc. Dallas TX USA; ^3^ Department of Epidemiology Rollins School of Public Health Emory University Atlanta GA USA

**Keywords:** HIV self‐testing, social networks, costs and cost‐effectiveness, costing methods, clinical trial expenditures, United States

## Abstract

**Introduction:**

HIV testing is an essential prerequisite for accessing treatment with antiretroviral therapy or prevention using pre‐exposure prophylaxis. Internet distribution of HIV self‐tests is a novel approach, and data on the programmatic cost of this approach are limited. We analyse the costs and cost‐effectiveness of a self‐testing programme.

**Methods:**

Men who have sex with men (MSM) reporting unknown or negative HIV status were enrolled from March to August 2015 into a 12‐month trial of HIV self‐testing in the United States. Participants were randomly assigned either to the self‐testing arm or the control arm. All participants received information on HIV testing services and locations in their community. Self‐testing participants received up to four self‐tests each quarter, which they could use themselves or distribute to their social network associates. Quarterly follow‐up surveys collected testing outcomes, including number of tests used and new HIV diagnoses. Using trial expenditure data, we estimated the cost of implementing a self‐testing programme. Primary outcomes of this analysis included total programme implementation costs, cost per self‐test completed, cost per person tested, cost per new HIV diagnosis among those self‐tested and cost per quality adjusted life year (QALY) saved.

**Results:**

A total of 2665 men were assigned either to the self‐testing arm (n = 1325) or the control arm (n = 1340). HIV testing was reported by 971 self‐testing participants who completed a total of 5368 tests. In the control arm, 619 participants completed 1463 HIV tests. The self‐testing participants additionally distributed 2864 self‐tests to 2152 social network associates. Testing during the trial identified 59 participants and social network associates with newly diagnosed HIV infection in the self‐testing arm; 11 control participants were newly diagnosed with HIV. The implementation cost of the HIV self‐testing programme was $449,510. The cost per self‐test completed, cost per person tested at least once, and incremental cost per new HIV diagnosis was $61, $145 and $9365 respectively. We estimated that self‐testing programme potentially averted 3.34 transmissions, saved 14.86 QALYs and nearly $1.6 million lifetime HIV treatment costs.

**Conclusions:**

The HIV self‐testing programme identified persons with newly diagnosed HIV infection at low cost, and the programme is cost saving.

## 1 Introduction

An estimated 1.1 million people in the United States are living with HIV, and 14% are unaware of their HIV‐positive status 1. Gay, bisexual and other men who have sex with men (MSM) have a higher HIV prevalence, incidence and number of undiagnosed infections than any other group 2. Surveillance data from the Centers for Disease Control and Prevention (CDC) show that HIV incidence decreased in the overall U.S. population in recent years, but incidence among MSM remained constant or increased among some age groups, including men aged 25 to 34 years and ≥55 years, and one racial/ethnic group, Hispanic MSM 2. The estimated percentage of HIV infections that were undiagnosed in 2016 was high among MSM overall (16.4%), and especially among Asian (20.5%), Hispanic (20.2%) and African American (19.2%) MSM 1.

Identifying HIV infections through HIV testing is the critical first step in moving persons with HIV into the HIV care continuum, including treatment and viral suppression that enhance survival and quality of life, and prevent HIV transmission 3, 4, 5, 6. The CDC recommends that all sexually active MSM be screened for HIV at least annually, and that clinicians should consider the potential benefits of more frequent screening (e.g. every three or six months) based on their patients' individual risk factors and local HIV epidemiology and policies 7. The CDC's 2016 nonclinical testing guidelines suggested the use of novel testing strategies, such as HIV self‐testing 8. HIV self‐testing can reduce barriers to testing and potentially be delivered at low cost 8, 9.

In 2012, the U.S. Food and Drug Administration (FDA) approved the first HIV self‐test for use in the United States, the OraQuick In‐Home HIV Test (*OraQuick*; OraSure Technologies, Inc.), a rapid test that provides results in 20 minutes 10. Although HIV self‐testing is relatively new in the United States, it has generated increasing interest worldwide 11, 12. The World Health Organization strongly recommended that HIV self‐testing be offered as an additional approach to clinical and targeted HIV testing services 11. In the United States, MSM reported a high willingness to use HIV self‐tests 13, 14, and using self‐tests results in increased screening and awareness of HIV status 14, 15. However, the cost‐effectiveness of this strategy for MSM has not been established.

To assess the feasibility and potential benefits of HIV self‐testing among MSM, CDC sponsored a nationwide randomized controlled trial (RCT), Evaluation of Rapid HIV Self‐Testing among MSM Project (eSTAMP: NCT02067039), in the United States 13, 15. In this analysis, we use trial expenditure data to estimate the cost and cost‐effectiveness of delivering a self‐testing programme, potentially by a health department or community‐based organization, in a non‐research setting 16, 17, 18, 19. Although the testing would be performed by participants, a self‐testing programme would likely require an organization to conduct recruitment, ship test kits, and collect and report data.

## 2 Methods

### 2.1 Study design and participants

The eSTAMP project investigators provided detailed expenditure data on the trial in the United States, including the development and implementation phases, which began in October 2014 and ended in September 2016 (Tables S1 and S2). Trial implementation began with the recruitment of participants who resided in the United States from March through August 2015 via banner advertisements placed on internet sites, including Facebook, other social network sites, and music and dating sites serving MSM. Eligible participants who reported never having been diagnosed with HIV infection were enrolled in the 12‐month longitudinal study. Study participants randomly assigned to the self‐testing arm were sent by FedEx four HIV self‐tests (2 OraQuick oral fluid tests; 2 SURE CHECK HIV 1/2 Assay (*Sure Check*; Chembio Diagnostics System, Inc.) finger‐stick blood tests). Participants could request up to four additional self‐tests to replace the ones they had used or given away, after completing each of the follow‐up online surveys at three, six and nine months. Participants could use the self‐tests or distribute them to their social network associates, for example, friends or sexual partners. The participants could report their self‐test results online through the study website or by phone. A participant who reported a preliminary positive self‐test result was considered to have a new diagnosis unless subsequent, confirmatory testing indicated a false‐positive result 15. All study participants and social network associates who used the study HIV self‐tests were able to call a toll‐free hotline to talk with a counsellor from 9 AM to 5 PM Eastern Time, Monday–Friday, and a toll‐free crisis line after‐hours and on weekends. The eSTAMP study website included a link to AIDSVu.org, where all study participants could locate information on local HIV testing services in the community. Participants assigned to the control arm were given access to the eSTAMP website with a link to AIDSVu.org, and they were not given self‐tests by the eSTAMP study. Details on the trial protocol are reported elsewhere 15.

### 2.2 Costs and cost‐effectiveness analysis

We used two steps to estimate self‐testing programme implementation costs over 12‐month period, based on invoices from the trial implementation phase. Full trial expenditures, including those for development and implementation, are reported in Table S1. First, we identified the types of resources from the trial implementation likely to be part of the testing strategy's actual programme implementation. Second, we estimated the fraction of the expenditures for those resources that would be required in the implementation (i.e. excluding research costs). Co‐authors involved in the trial as Project Officers (RM, PC), Principal Investigator (PS) and Project Manager (MN) generated the estimates for both steps. Based on the number and scope of activities expected to be associated with programme implementation, we developed a total economic cost of the programme and, within total costs, we estimated fixed (likely to be incurred regardless of the number of participants) and variable (likely to increase or decrease according to the number of participants) costs. The fixed costs included internet site design and monitoring for participants recruitment, programme administration, and office overhead, and the variable costs included HIV test kits, mailing test kits and supplies, and incentives to participants. We excluded the costs related to research and development.

Based on the total programme implementation cost, we estimated the average cost per test completed, cost per person tested and the incremental cost per new HIV diagnosis. We calculated costs per outcome by dividing the total cost by the number of self‐tests completed (assuming that all self‐tests distributed to social network members were used), by the number of persons (participants and social network members) who completed at least one self‐test, and by the number of new HIV diagnoses over and above those in the control arm. We assumed that all costs associated with the self‐testing programme were in addition to the costs of standard‐of‐care HIV testing services received by control participants.

Using the additional new HIV diagnoses identified under self‐testing arm, we estimated the incremental number of HIV transmissions averted, lifetime HIV treatment costs saved, QALYs saved and cost per QALY saved 20, 21, 22. We assumed a reduction in transmission per new HIV diagnosis (0.0696), based a lower HIV transmission rate (0.0516) attributable to MSM being aware of their HIV status compared with those unaware of their status (0.1212) 23. We applied 4.45 quality adjusted life years (QALYs) saved per infection averted, based on the patients who were diagnosed and entered HIV care at a CD4 count of 500 cells/mL or above 5. We assumed the lifetime HIV treatment cost to be $466,000 per infection averted 5, and cost‐effectiveness threshold to be $100,000, commonly used in the cost‐effectiveness analysis in the United States 24, 25, 26.

We estimated costs and cost‐effectiveness of the self‐testing programme from a health care provider's perspective since we did not measure participant‐related costs 16, 27, 28. Costs are reported in 2016 U.S. dollars.

### 2.3 Sensitivity analysis

We conducted sensitivity analyses on costs and outcomes to explore how our cost‐effectiveness findings might change under different assumptions about programme services and the length of the programme. First, we streamlined the fixed costs by assuming that the self‐testing programme could be an addition to HIV testing services already being provided by the agency, thus generating only minimal additional fixed costs. For instance, we halved the time required for project administration, to approximate the time a project manager would actually spend delivering the self‐testing programme. Furthermore, we excluded office space and overhead costs while retaining participant recruitment and monitoring costs and all variable costs. Second, we shortened the programme duration to six and three months, instead of 12. For this analysis, we assumed that recruitment and monitoring costs would remain the same as in the base case, but that other fixed and variable costs would be incurred only for the length of the intervention. Third, we explored cost‐effectiveness when the number of new diagnoses associated with self‐testing in a given timeframe increased and decreased.

The trial was approved by the Institutional Review Board at Emory University, Atlanta, Georgia. Sure Check was used under an Investigational Device Exemption from the FDA. All participants provided separate online consent for screening and participation in the study, and they could voluntarily withdraw during the study.

## 3 Results

A total of 2665 men were randomly assigned to either the self‐testing arm (n = 1325) or the control arm (n = 1340, Table 1) and completed a baseline survey. During the 12‐month trial period, HIV testing was reported by 971 self‐testing participants (two of whom did so by calling the study hotline only) and 619 control participants. Control participants reported testing 1463 times and self‐testing participants reported testing 5368 times, of which 4504 were with self‐tests received from the study. The self‐testing participants distributed 2864 self‐tests to 2152 social network associates. Testing during the trial identified 25 new HIV diagnoses among self‐testing participants, 34 new HIV diagnoses among their social network associates, and 11 new diagnoses among control participants. Thus, the self‐testing arm yielded a total of 59 new HIV diagnoses among the 3477 persons who received a study HIV self‐test, or 48 more than the control arm.

**Table 1:** Health outcomes and costs of HIV self‐testing programme based on a randomized controlled trial, 2015 to 2016

	HIV self‐testing	Control
Participants		
Participants enrolled in the trial	1325	1340
Participants completing at least 1 follow‐up survey	1014	977
Participants completing any HIV test^a^	971	619
Participants completing HIV self‐test	938	–
No. of all HIV tests reported by participants	5368	1463
No. of HIV self‐tests reported by participants	4504	–
Participants with new HIV diagnosis	25	11
Social network associates		
No. of self‐tests distributed to social network associates	2864	–
Social network associates using self‐tests	2152	–
Social network associates with new HIV diagnosis	34	–
No. of additional new HIV diagnoses under self‐testing arm (N)	48	–
Programme costs^b^		
Total cost (C)	$449,510	–
Cost per self‐test completed^c^	$61	–
Cost per person tested^c^	$145	–
Incremental cost‐effectiveness^d^		
Transmissions averted per new HIV diagnosis (t)^e^	0.0696	–
QALYs saved per transmission averted (Q)^f^	4.4500	–
No. of transmissions averted (A = tN)	3.34	–
No. of QALYs saved (AQ)	14.86	–
Lifetime HIV treatment cost saved per transmission averted (T)^g^	$466,000	–
Total lifetime treatment cost saved (AT)	$1,556,454	–
Cost per new HIV diagnosis (C/N)	$9365	–
Cost per HIV transmission averted (C/A)	$134,583	–
Cost per QALY saved (C‐AT)/AQ^h^	($74,476)	–

a Included two participants who reported their HIV diagnoses by telephone;

b costs exclusively related to research and development were excluded. Costs are reported in 2016 U.S. dollars;

c average cost per self‐test completed was calculated by dividing the total programme cost by the number of self‐tests completed by the participants (n = 4504) and their social network associates (n = 2864), and assumed all self‐tests distributed to the associates were used. The cost per person tested was calculated by dividing the total programme cost by the number of participants completing self‐test (938) and the associates using self‐tests (2152);

d incremental cost‐effective ratios (ICER) defined as, cost per new diagnosis (C/N), cost per HIV transmission averted (C/A), and cost per QALY saved = [(C‐AT)/AQ] ≤ $100,000, where C is total programme cost, N is additional new HIV diagnoses, A is no. of transmissions averted, T is lifetime HIV treatment cost saved per transmission averted, and Q is no. of quality adjusted life years (QALYs) saved per transmission averted 21, 22, 23; all costs and health outcomes are additional to those of the controlled arm. Threshold for cost‐effectiveness assumed to be $100,000 24, 25, 26. Cost saving threshold: ICER = [(C−AT)/AQ] < 0, or (C−AT) <0, or C < AT;

e transmissions averted per new HIV diagnosis (t, 0.0696) is based on the estimated transmissions attributable to MSM aware of their HIV status (0.0516) compared with those unaware of their status (0.1212) 23;

f QALYs saved per transmission averted (Q) is based on the patients diagnosed and entered HIV care at a CD4 count of 500 cells/mL or above 5;

g Lifetime treatment cost saved per HIV transmission averted (T) based on the patients diagnosed and entered HIV care at a CD4 count of 500 cells/mL or above, discounted at 3%^5^, and adjusted to 2016 US dollars;

h result in parentheses shows a negative ICER, indicating the programme is cost saving to the health care provider, that is, the self‐testing programme cost (C) was less than the cost of estimated HIV transmissions averted by the programme (AT). The cost per new diagnosis can be as high as $32,400 (i.e. $1,556,500/48) for cost saving threshold, and $63,400 for cost‐effectiveness, at $100,000 cost per QALY threshold.

We estimated the cost of the HIV self‐testing programme to be $449,510 (Tables 1 and 2). The cost per self‐test completed was $61, and the cost per person completing at least one self‐test was $145. When we subtracted the number of new diagnoses in the control arm from the new HIV diagnoses in the self‐testing arm, the incremental cost per new diagnosis (or incremental cost‐effectiveness ratio, ICER) was $9365. We estimated that the self‐testing programme averted 3.34 HIV transmissions, and saved 14.86 QALYs and $1,556,454 lifetime HIV treatment costs, much higher than the total programme cost ($449,510, Table 1), suggesting that the self‐testing programme was cost saving.

**Table 2:** HIV self‐testing programme costs and required resources based on a randomized controlled trial, 2015 to 2016

	HIV self‐testing programme cost ($)^a^	Distribution of cost (%)
Fixed costs		
Internet site design and monitoring		
Internet site design and monitoring	33,060	7.4%
Online advertising and recruitment	11,221	2.5%
Administration		
Project director/supervisor	543	0.1%
Project manager	45,036	10.0%
Administrative manager	475	0.1%
Data analysts	11,190	2.5%
Data cleaning and management	6978	1.6%
Internet technology security specialist	4489	1.0%
Clerical – Test kits shipping and handling	40,480	9.0%
Office overhead		
Office space (39.09% of labour cost)	43,676	9.7%
General and administrative overhead (16.18% of labour and test kits and supplies)	24,734	5.5%
Sub‐total	221,882	49.4%
Variable costs		
HIV testing kits distribution		
HIV test kits (N = 8654, $18.65/test)^b^	161,433	35.9%
Mailing test kits and supplies	36,161	8.0%
Incentives to participants	30,034	6.7%
Sub‐total	227,628	50.6%
Total	449,510	100.0%

a Cost refers to the total amount spent during the implementation phase of the trial on activities and resources that the investigators (co‐authors: RM, PC, PS, MN) determined would be required in the implementation of a self‐testing programme. Costs were reported in 2016 U.S. dollars;

b self‐test kits included those used by participants (n = 4504) and associates (n = 2864) and those not accounted for (n = 1286). OraQuick in‐home HIV test was approved by the U.S. Food and Drug Administration (FDA) for self‐test, and price of the test was negotiated between the project contractor and manufacturer, OraSure Technologies, Inc.; Sure Check was used under an Investigational Device Exemption from the FDA, and cost included the production, shipping and handling.

In our analysis, the key programme implementation resources identified are listed in Table 2. The fraction of the trial implementation expenditure that a programme would be expected to incur ranged from 0.05 (administrative manager) to 1.00 (e.g. test kits, N = 8654) of the trial implementation expenditures (Table S1). The distribution of the costs across programme activities showed that approximately half of the total costs were variable (Table 2). Among the fixed costs was recruitment of participants through online venues. Online dating and internet radio sites recruited the majority (95%) of the participants, and the cost per participant completing a baseline interview ranged from $2 to $123, depending on the recruitment site (Table S3).

**Figure 1:**
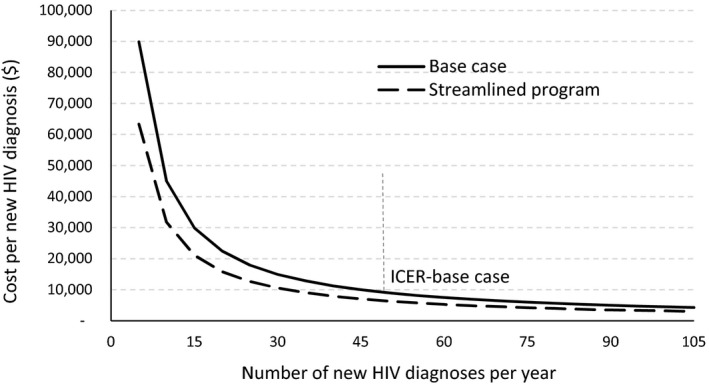
Relationship between incremental cost and new HIV diagnoses in self‐testing intervention.

In sensitivity analysis, when we assumed streamlined testing and reduced the fixed costs but included all variable costs in the analysis, the total annual programme cost decreased by 29% from the base case. The resulting average cost per self‐test completed, cost per person tested, and incremental cost per new diagnosis were estimated to be $43, $103 and $6603 (Table 3). We assumed the same number of new HIV diagnoses under streamlined testing as in the base case. Thus, the line showing relationship between new diagnoses and costs shifted down from the base case (Figure 1). When we reduced the programme length to six months and three months, the total cost decreased by 22% and 36% respectively, and the resulting average cost per self‐test completed, cost per person tested, and incremental cost per new diagnosis were $50 and $44, $171 and $200, and $11,288 and $13,618 respectively. These costs were based on the actual number of participants and associates who self‐tested and were diagnosed in the six‐ and three‐month periods: 2044 (34 new diagnoses) and 1431 (23 new diagnoses) respectively. In each of these sensitivity analyses, the programme remained cost saving.

**Table 3:** Sensitivity analysis of HIV self‐testing programme costs and cost‐effectiveness results

	Persons tested or self‐tested^a^	Test kits used^b^	New HIV diagnoses	Trans‐missions averted^c^	QALYs saved^c^	Total programme cost ($)^b^	Cost/self‐test ($)	Cost/person tested ($)^d^	Incr. cost/new diagnosis ($)^e^	Incr. cost/trans‐mission averted ($)^e^	Incr. cost/QALY saved ($)^e^
Twelve‐month programme: base case											
Self‐testing arm	3090	7368	59	3.34	14.86	449,510	61	145	9365	134,583	(74,476)
Control arm	619	–	11	–	–	–	–	–	–	–	–
Twelve‐month intervention: streamlined testing^f^											
Self‐testing arm	3090	7368	59	3.34	14.86	316,945	43	103	6603	94,893	(83,395)
Control arm	619	–	11	–	–	–	–	–	–	–	–
Six‐month intervention											
Self‐testing arm	2044	7061	34	2.37	10.53	349,932	50	171	11,288	147,909	(71,481)
Control arm	452	–	3	–	–	–	–	–	–	–	–
Three‐month intervention											
Self‐testing arm	1431	6462	23	1.60	7.12	285,976	44	200	13,618	178,687	(64,565)
Control arm	284	–	2	–	–	–	–	–	–	–	–

a Number of persons tested under the self‐testing arm (n = 3090) included the participants (n = 938) and associates (n = 2152) completing HIV self‐tests. The number of self‐tests (n = 7368) used included the tests used by participants (n = 4504) and associates (n = 2864). Of the total tests distributed (N = 8654), 1286 tests were not accounted for. All tests distributed to associates were assumed to have been used and, thus, all associates were assumed to have self‐tested at least once;

b six‐ and three‐month programmes included full recruitment and monitoring costs, but included fixed costs incurred only for the length of the intervention and variable costs based on the number of participants served during the intervention. While the length of these scenarios was assumed, self‐testing outcomes were based on actual data from the eSTAMP intervention;

c transmissions averted calculation is based on the estimated transmissions attributable to MSM aware of their HIV status (0.0516) compared with those unaware of their status (0.1212) 23, thus the difference is transmissions averted per new HIV diagnosis (0.0696). QALYs saved per transmission averted is based on the patients diagnosed and entered HIV care at a CD4 count of 500 cells/mL or above 5;

d cost per self‐test completed in six‐ and three‐month programmes were lower compared to the base case, partly because of the relatively larger number of tests distributed during those periods;

e Incr. is incremental, and incremental cost‐effective ratios (ICER) defined as, cost per new diagnosis (C/N), cost per HIV transmission averted (C/A), and cost per QALY saved [(C‐AT)/AQ] ≤$100,000, where C is total programme cost, N is additional new HIV diagnoses, A is no. of transmissions averted, T is lifetime HIV treatment cost saved per transmission averted, and Q is no. of quality adjusted life years (QALYs) saved per transmission averted 21, 22, 23;

f streamlined self‐testing programme excluded fixed costs, except participant recruitment and monitoring, and halved the project administration time.

## 4 Discussion

We estimated the total cost of the HIV self‐testing programme to be $449,510 with the programme reaching and providing self‐tests to 3477 persons (participants and social network associates). The average cost per self‐test completed, cost per person tested and incremental cost per new HIV diagnosis were $61, $145 and $9365 respectively. The self‐testing programme averted 3.34 HIV transmissions, and saved 14.86 QALYs and nearly $1.6 million lifetime HIV treatment costs. Thus, the programme was cost saving. Cost analysis of HIV testing in various health care and non‐health care settings under the CDC's Advancing HIV Prevention project showed that the cost per new HIV diagnosis ranged from $3500 to $36,300 ($US 2016) 29. Similarly, the cost analysis of the Expanded Testing Initiative showed that the cost per new HIV diagnosis ranged from $6100 to $73,600 ($US 2016) 30. Cost‐effectiveness modelling of HIV testing interventions that incorporated longer‐term costs and health consequences, such as lifetime HIV treatment costs and quality‐adjusted life‐years (QALYs), suggests that the cost per new HIV diagnosis can be as high as $28,300 ($US 2016) and remain cost saving 22. Our analysis shows that the cost per new diagnosis can be as high as $32,400 for the self‐testing programme to remain cost saving and $63,400 to remain cost effective, at $100,000 cost per QALY threshold.

The low cost per new HIV diagnosis of the self‐testing programme is related to the number of newly diagnosed infections reported among self‐testing participants and social network associates (n = 59). Furthermore, even participants in the control arm had reported positive test results that were considered newly diagnosed infections (n = 11) given that they started the RCT as HIV negative or HIV unknown. This suggests that the internet‐based recruitment strategies used in the trial were successful in reaching persons at risk of HIV infection and engaging them in testing. The number of infections among persons who received a self‐test (59/3477, 1.7%) is much higher than those detected in recent large‐scale HIV testing programmes implemented by health departments in health care (median, 0.3%) and non‐health care (median, 0.6%) settings 31, and higher or within the range reported in HIV screening programmes in jails (0.2% to 1.3%) 32, 33, 34 and targeted HIV testing programmes implemented by community‐based organizations in outreach settings (0.7% to 2.2%) 35, 36.

Our analysis showed a relatively high proportion of fixed costs (50% of the total) in the self‐testing programme, which is common in other HIV testing programmes 16, 29, 35. We explored the potential impact of reduced fixed costs by streamlining the self‐testing programme, and when we did so, the total annual programme cost decreased by 29% from the base case, suggesting that there may be room for greater programme efficiencies. Furthermore, self‐testing programmes may be able to reduce fixed or variable costs by collaborating with test manufacturers for delivery of some of the services, including direct shipping of rapid self‐test kits to participants. The New York City Health Department has already initiated such a collaborative programme with manufacturers 37. We also explored the impact of shorter programme periods (six and three months). The results showed that the average cost per person tested and incremental cost per new HIV diagnosis (i.e. ICER) might increase with shorter programme periods; however, the ICERs were still much lower than the cost saving threshold 20.

Prior research documents a willingness to self‐test 13, 38, 39. Nunn and colleagues showed that among the participants surveyed in predominantly African American U.S. neighbourhoods with 3% HIV prevalence, nearly 90% were likely or very likely to accept a free HIV self‐test, and 55% were willing to purchase a test; however, only 23% were willing to pay the market price ($40/test) 38. Therefore, public sector funding may be critical for the adoption of HIV self‐testing among high‐risk populations.

### 4.1 Limitations

Limitations of our study include that we conducted the cost analysis retrospectively based on trial expenditures reported to the CDC. Hence, the initial cost data reflected expenditures inclusive of research and development costs. Although we developed a systematic approach to estimate the cost of the implementation of a self‐testing programme exclusive of research and development costs, we may have under‐ or overestimated actual implementation costs.

Second, all HIV self‐testing and clinic based testing information was based on participants' reporting their HIV test results online or by phone to study staff. We assumed the reported HIV‐positive test result was a new diagnosis unless subsequent, confirmatory testing indicated a false‐positive result 15. We were unable to confirm all reported positive test results; thus the number of new HIV diagnoses in our analysis could be lower and the ICER higher than those reported in the base case (Figure 1). In addition, some studies have found that only about 50% to 60% of positive HIV tests reflect new diagnoses 40, 41, 42. However, all eSTAMP participants reported never having been diagnosed with HIV and 17% reported never having been tested for HIV 15, reducing the likelihood of a previous diagnosis.

Third, the average cost of an HIV test kit was $18.65 in our analysis. The price of OraQuick in‐home HIV test, the FDA approved self‐test, was negotiated between the project contractor and manufacturer (OraSure Technologies, Inc.). Sure Check was used under an investigational device exemption from the FDA, and the cost included the production of the device, shipping and handling. If an organization implementing a HIV self‐testing programme were required to pay higher price than the price used in our analysis, ICER would be higher and self‐testing programme would be less cost‐effective.

Fourth, some study participants may have been unwilling to disclose their positive test results. The eSTAMP trial did not receive any follow‐up information from 25% of the participants who were mailed the HIV self‐testing kits after completing the baseline survey 15. If more HIV infections were diagnosed than reported, the ICER could be lower, making the self‐testing programme even more cost‐effective (Figure 1).

## 5 Conclusions

We estimated the costs and cost‐effectiveness of a rapid HIV self‐testing programme based on the expenditure data reported by a randomized controlled trial in the United States. Our analysis showed the potential for an HIV self‐testing programme to be cost saving in the base case and under several scenarios. Making HIV testing simple, accessible, and routine, and increasing the number of people who know their diagnosis is key to preventing HIV transmission and supports the recently announced U.S. initiative, “Ending the HIV Epidemic: A Plan for America” 6. HIV self‐testing, through internet recruitment and the distribution of tests by mail, could be a promising new strategy to reach more at‐risk persons at a relatively low cost. A self‐testing programme also could serve those who cannot or will not access traditional HIV testing services.

## Competing Interests

The authors declare no competing interests.

## Authors' Contributions

All authors contributed to the conceptualization of the paper. RK Shrestha, RJ MacGowan and PR Chavez wrote the first draft of the paper. SL Sansom, JH Mermin, PS Sullivan and M Nobel contributed to subsequent drafts of the paper. RJ MacGowan, PR Chavez and RK Shrestha had full access to all the data in the study and take responsibility for the integrity of the data and the accuracy of the data analysis. All authors approved the final draft of the paper.

## Supporting information


**Table S1.** eSTAMP trial expenditures and estimated costs of the self‐testing programme, 2015 to 2016.
**Table S2.** eSTAMP trial staff time and allocation of labour hours to the self‐testing programme, 2015 to 2016.
**Table S3.** Advertising and recruitment cost of the an HIV self‐testing programme based on a randomized controlled trial, 2015 to 2016.
