# Supplementary material for: Estimating the costs and cost‐effectiveness of HIV self‐testing among men who have sex with men, United States
Source: J Int AIDS Soc. 2020 Jan 20;23(1):e25445. doi: 10.1002/jia2.25445 (PMC6970935; doi:10.1002/jia2.25445)
Supplement: Supplementary file 1 — Table S1. eSTAMP trial expenditures and estimated costs of the self‐testing programme, 2015 to 2016. Table S2. eSTAMP trial staff time and allocation of labour hours to the self‐testing programme, 2015 to 2016. Table S3. Advertising and recruitment cost of the an HIV self‐testing programme based on a randomized controlled trial, 2015 to 2016. [file JIA2-23-e25445-s001.docx]

**Appendix Table 1. The eSTAMP trial expenditures and estimated costs of the self-testing program, 2015–2016**

|  | **Total Trial Spending^a^** | | | | **Trial Development^b^** | | | | **Trial Implementation^b^** | | | | **Self-Testing Program^c^** | | | |
| --- | --- | --- | --- | --- | --- | --- | --- | --- | --- | --- | --- | --- | --- | --- | --- | --- |
|  | **EXP ($)** | | **Percent** | | **EXP ($)** | | **Percent** | | **EXP ($)** | | **Percent** | | **Allocation** | | **Cost ($)** | |
| **Trial start-up and recruitment** |  | |  | |  | |  | |  | |  | |  | |  | |
| Design & production of home testing kit box | 66,106 | | 2.5% | | 35,718 | | 54.0% | | 30,388 | | 46.0% | | 0.00 | | --- | |
| Internet certification, site design, and monitoring | 552,168 | | 20.9% | | 221,568 | | 40.1% | | 330,600 | | 59.9% | | 0.10 | | 33,060 | |
| Online advertising, recruitment, and market research | 174,995 | | 6.6% | | 152,500 | | 87.1% | | 22,495 | | 12.9% | | 0.50 | | 11,221 | |
| **Trial administration^d^** |  | |  | |  | |  | |  | |  | |  | |  | |
| Project director/supervisor | 46,594 | | 1.8% | | 41,161 | | 88.3% | | 5,433 | | 11.7% | | 0.10 | | 543 | |
| Project manager | 219,968 | | 8.3% | | 69,847 | | 31.8% | | 150,121 | | 68.2% | | 0.30 | | 45,036 | |
| Administrative manager | 20,243 | | 0.8% | | 10,746 | | 53.1% | | 9,497 | | 46.9% | | 0.05 | | 475 | |
| Data analysts | 65,818 | | 2.5% | | 9,867 | | 15.0% | | 55,951 | | 85.0% | | 0.20 | | 11,190 | |
| Data cleaning and management | 102,625 | | 3.9% | | 32,849 | | 32.0% | | 69,776 | | 68.0% | | 0.10 | | 6,978 | |
| Internet technology security specialist | 44,886 | 1.7% | | 22,443 | | 50.0% | | 22,443 | | 50.0% | | 0.20 | | 4,489 | |  |
| Trial monitoring and follow-up | 435,561 | 16.5% | | 100,000 | | 23.0% | | 335,561 | | 77.0% | | 0.00 | | - | |  |
| Clerical: shipping and handling | 55,920 | 2.1% | | 15,440 | | 27.6% | | 40,480 | | 72.4% | | 1.00 | | 40,480 | |  |
| **HIV testing kits and supplies** |  |  | |  | |  | |  | |  | |  | |  | |  |
| HIV self-tests^e^ | 161,433 | 6.1% | | -- | | -- | | 161,433 | | 100.0% | | 1.00 | | 161,433 | |  |
| Mailing of test kits^f^ | 99,135 | 3.7% | | 62,975 | | 63.5% | | 36,161 | | 36.5% | | 1.00 | | 36,161 | |  |
| Incentives | 165,170 | 6.2% | | 15,000 | | 9.1% | | 150,170 | | 90.9% | | 0.20 | | 30,034 | |  |
| Conference calls | 331 | 0.0% | | 245 | | 74.2% | | 85 | | 25.8% | | 0.00 | | - | |  |
| Travel and transportation | 2,237 | 0.1% | | 2,120 | | 94.8% | | 117 | | 5.2% | | 0.00 | | - | |  |
| **Office space and other overhead** |  |  | |  | |  | |  | |  | |  | |  | |  |
| Office space (39.09% of labor costs)^g^ | 103,352 | 3.9% | | 45,892 | | 44.4% | | 57,460 | | 55.6% | | -- | | 43,676 | |  |
| G & A overhead (16.18% of labor and test kits and supplies)^h^ | 111,106 | 4.2% | | 31,562 | | 28.4% | | 79,544 | | 71.6% | | -- | | 24,734 | |  |
| Sub-contract handling fee (3.15% of sub-contract cost) | 43,702 | 1.7% | | 17,579 | | 40.2% | | 26,123 | | 59.8% | | 0.00 | | - | |  |
| Other fixed fees (7.0% of total cost) | 172,994 | 6.5% | | 63,697 | | 36.8% | | 109,298 | | 63.2% | | 0.00 | | - | |  |
| **Total^i^** | 2,644,343 | 100.0% | | 951,208 | | 36.0% | | 1,693,136 | | 64.0% | | --- | | 449,510 | |  |

^a^ Total Trial Spending includes trial development and implementation expenditures. EXP represents trial expenditures. Percent represents distribution of the total expenditure (i.e., column percent).

^b^ Trial Development represents the period after the pilot and before the trial launch (October 2014-March 2015). Trial Implementation represents the period of the trial (April 2015-September 2016), including the 12-month randomized controlled trial. Percent reflects the proportion of the total expenditure for that activity or resource (i.e., row percent).

^c^ Cost refers to the total amount spent during the implementation phase of the trial on activities and resources that the investigators (co-authors: RM, PC, PS, MN) determined would be required in the implementation of a self-testing program. Allocation shows the fraction of the total implementation expenditures for each resource expected in the implementation.

^d^ Labor costs were calculated based on the labor hours and wage rates reported with personnel expenditure data; 35% fringe benefits were incorporated into the wage rates during analysis (BLS).

^e^ Participants assigned to the self-testing arm initially received four rapid HIV self-tests: 2 OraQuick tests, 2 Sure Check tests; they had the option of requesting additional tests every 3 months to replace those they had used or given away.

^f^ Expenditures reported under Trial Development included delayed claims that were incurred earlier in the formative phase of the project. Approximately $57,500 were billed within 2 months into the trial development phase.

^g^ Office space cost was calculated as 39.09% of the labor cost, without fringe benefits; the cost allocation was based on the total home office cost (74.09% of labor cost) reported in the original billing data. In cost analysis, the home office costs were allocated to office space (39.09%) and fringe benefits (35.0%).

^h^ General and administrative overhead was calculated as 16.18% of the administration cost (without fringe benefits) and HIV testing kits and supplies cost.

^i^ In addition, the project billed $4,078,343 for the formative activities of the study (end data: February 2014) prior to the development phase.

All expenditures were compiled based on the project billing data, and reporting of some expenditures may have been delayed because of administrative reasons.

**Appendix Table 2. The eSTAMP trial staff time and allocation of labor hours to the self-testing program, 2015–2016**

|  | **Total Trial Spending^a^** | | **Trial Development^b^** | | **Trial Implementation^b^** | | **Self-Testing Program^c^** | |
| --- | --- | --- | --- | --- | --- | --- | --- | --- |
|  | **Hours** | **Percent** | **Hours** | **Percent** | **Hours** | **Percent** | **Allocation** | **Hours** |
| Project director/supervisor | 506 | 2.7% | 447 | 88.3% | 59 | 11.7% | 0.10 | 6 |
| Project manager | 2,692 | 14.4% | 855 | 31.8% | 1,837 | 68.2% | 0.30 | 551 |
| Administrative manager | 324 | 1.7% | 172 | 53.1% | 152 | 46.9% | 0.05 | 8 |
| Data analysts | 1,448 | 7.8% | 217 | 15.0% | 1,231 | 85.0% | 0.20 | 246 |
| Data cleaning and management | 2,257 | 12.1% | 722 | 32.0% | 1,535 | 68.0% | 0.10 | 153 |
| Internet technology security specialist | 641 | 3.4% | 321 | 50.0% | 321 | 50.0% | 0.20 | 64 |
| Trial monitoring and follow-up | 9,580 | 51.3% | 2,199 | 23.0% | 7,380 | 77.0% | 0.00 | 0 |
| Clerical: shipping and handling | 1,230 | 6.6% | 340 | 27.6% | 890 | 72.4% | 1.00 | 890 |
| **Total** | 18,677 | 100.0% | 5,273 | 28.2% | 13,405 | 71.8% |  | 1,919 |

^a^ Total Trial Spending includes staff time spent on trial development and implementation. Percent represents the distribution of the total hours (i.e., column percent).

^b^ Trial Development represents the period after the pilot and before the trial launch (October 2014-March 2015). Trial Implementation represents the period of the trial (April 2015-September 2016), including the 12-month randomized controlled trial. Percent reflects the proportion of the total hours for that activity (i.e., row percent).

^c^ Hours refers to the total staff time spent during the implementation phase of the trial on activities that the investigators (co-authors: RM, PC, PS, MN) determined would be required in the implementation of a self-testing program. Allocation shows the fraction of the trial implementation hours for each staff/activity expected in the implementation.

**Appendix Table 3. Advertising and recruitment cost of the an HIV self-testing program based on a randomized controlled trial, 2015–2016**

|  | **Advertisements Displayed** | **Participants Completing Baseline Interview (%)** | **Total Cost ($)^a^** | **Cost per Person Interviewed ($)** |
| --- | --- | --- | --- | --- |
| Dating site 1 | 7,635,714 | 711 (24.3) | 24,500 | 34.46 |
| Dating site 2 | 5,000,000 | 960 (32.8) | 21,000 | 21.88 |
| Pandora internet radio site | 6,228,373 | 1,111 (37.9) | 87,000 | 78.31 |
| Gay Ad Network | 3,555,378 | 58 (2.0) | 7,111 | 122.60 |
| Facebook | 107,366 | 16 (0.5) | 637 | 39.82 |
| MUSED magazine | 10,000 | 72 (2.5) | 170 | 2.36 |
| **Total** | --- | 2,928 (100.0) | 140,418 | 47.96 |

^a^ Total amount paid to the internet sites for posting advertisements, based on the data available for each advertising site. Cumulative billing data reported in Appendix Table 1 showed $174,995 for online advertising and recruitment, which also included the trial development expenditures.
